# Pleural Effusion: Shedding Light on Pleural Disease Beyond Infection and Malignancy

**DOI:** 10.3390/medicina61030443

**Published:** 2025-03-03

**Authors:** William C. Harding, Abdul R. Halawa, Mazen M. Aiche, Bilal Zafar, Hyeon-Ju R. Ali, Lara Bashoura, Saadia A. Faiz

**Affiliations:** 1 Divisions of Pulmonary, Critical Care Medicine and Sleep Medicine, McGovern Medical School, University of Texas Health, Houston, TX 77030, USA; william.c.harding@uth.tmc.edu (W.C.H.); abdul.rahman.r.halawa@uth.tmc.edu (A.R.H.); mazen.m.aiche@uth.tmc.edu (M.M.A.); bilal.zafar@uth.tmc.edu (B.Z.); 2 Department of Cardiology, The University of Texas MD Anderson Cancer Center, Houston, TX 77030, USA; hrali@mdanderson.org; 3 Unit 1462, Department of Pulmonary Medicine, The University of Texas MD Anderson Cancer Center, P.O. Box 301402, Houston, TX 77030, USA; lbashoura@mdanderson.org

**Keywords:** pleural effusion, transudate, non-malignant pleural effusion, hepatic hydrothorax, heart failure, indwelling pleural catheter

## Abstract

*Background and Objectives*: Non-malignant pleural effusions (NMPEs) are the most frequently encountered pleural disease. They arise from various non-malignant, non-infectious clinical conditions, including cardiac, renal, and hepatic organ dysfunction. Despite their wide prevalence, there is a lack of literature for NMPE. This publication aims to provide an updated overview of the causes, diagnostic strategies, and management options for NMPE. *Materials and Methods*: This review synthesizes findings from studies published on NMPE, focusing on the presentation, diagnosis (such as imaging and pleural fluid analysis), and management strategies. Studies were selected based on relevance and were analyzed to provide a comprehensive summary of current practices. *Results*: The review highlights different etiologies of NMPE, including organ-specific factors. Imaging, pleural fluid analysis, and clinical correlation remain crucial in diagnosing the etiology of NMPE. Treatment strategies are largely dependent on the underlying condition. Medical management remains the mainstay for many causes. In some cases, interventions, such as thoracentesis, tunneled indwelling pleural catheter, or pleurodesis, are necessary. *Conclusions*: NMPE is a heterogeneous condition with a wide prevalence and significant implications. They present a diagnostic and management challenge due to patient complexity and evolving therapeutic options.

## 1. Introduction

**Figure 1:**
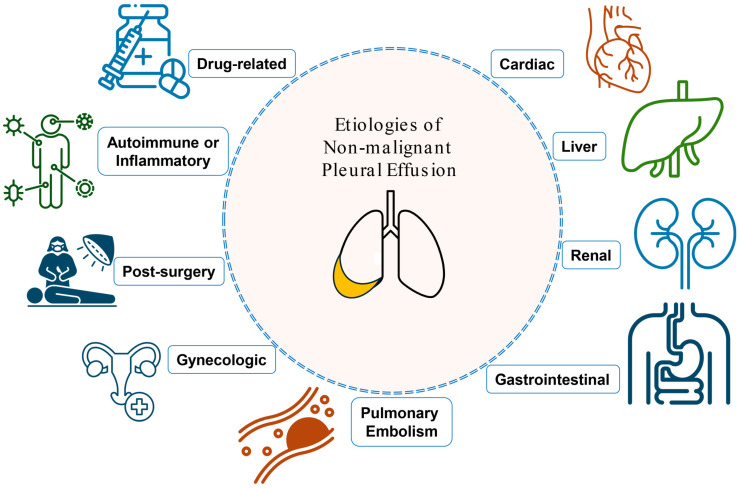
**Etiologies of non-malignant pleural effusions (NMPEs).** NMPEs are a significant contributor to healthcare expenditures, and they can be attributed to several different etiologies.

Non-malignant pleural effusions (NMPEs) represent the most frequently encountered pleural disease, and NMPEs may have significant consequences in terms of hospitalizations, healthcare costs, and mortality. In the United States, NMPE accounts for 63.5% of total hospitalizations related to adult pleural disease and costs the United States healthcare system USD 10.1 billion annually [1]. As the proportion of people with chronic disease has increased so too have incidence rates for NMPE, now estimated to inflict 252 per 100,000 persons [2]. Both the visceral and parietal pleurae play significant roles in maintaining normal homeostasis, and pleural fluid production is dependent on the balance of hydrostatic and oncotic pressure differences between the pleural space and systemic and pulmonary circulations [3]. Lymphatic vessels in the parietal pleura can escalate resorption by a factor of 20 in response to increases in pleural fluid, so the emergence of pleural effusion indicates higher production of fluid, reduced resorption, or a combination of the two [3,4]. Inextricably linked with organ dysfunction, common causes of non-infectious NMPE include cardiac, renal, and hepatic failure, and such organ dysfunction associated with NMPE carries an estimated 1-year mortality rate of as high as 50% [5]. Compared to infectious and malignant pleural effusions, the lack of literature for NMPE, increasing patient complexity, and paucity of therapeutic options have made proper diagnosis and management of NMPE challenging. In this review, we will provide an evidence-based update on the diagnosis, various etiologies of NMPE including organ-specific factors (Figure 1), and management strategies.

## 2. Diagnosis

The diagnosis for NMPE includes biochemical analysis, imaging, and clinical correlation. Biochemical analysis remains the gold standard for diagnosis of NMPE. Other characteristics, including gross appearance and odor, may also provide clues into underlying etiology. For example, opaque whitish fluid may be chylous, pseudochylous, or pus, or a urine smell may signify urinothorax. Classification of the fluid into transudate and exudate allows the generation of a differential diagnosis and guidance for further management decisions. Categorization of the pleural effusion aims to maximize identification of exudates to avoid misdiagnosis of serious conditions such as infection and malignancy. Statistically, the most common cause of exudative non-malignant pleural effusion in the developed world is parapneumonic effusion, and tuberculous pleurisy accounts for 30–80% of effusions in the developing world [6]. Excluding infections, exudative NMPEs represent a small portion of observed effusions; however, they carry significant disease implications. For example, an autoimmune-related pleuritis, which, if left untreated, can lead to fibrothorax, pancreatitis-associated effusion may significantly increase mortality, or drug-related pleural disease seen with amiodarone or methotrexate can add to symptom burden [7,8,9,10]. Light’s criteria are sensitive tests and very specific for exudates, but it may misclassify approximately 25% of transudates [11,12]. In fact, approximately 30% of effusions related to heart failure and 18% related to cirrhosis may be misclassified. Alternative rules may be used in other uncertain circumstances (Table 1); however, judicious clinical reasoning is paramount [13,14,15,16]. 

There are various imaging options to detect pleural effusions. Conventionally, an upright two-view chest radiograph is often the initial test of choice. With a meniscus on the lateral chest radiograph, pleural fluid volume can be estimated at 50 cc, and a meniscus on the posteroanterior radiograph, 200 mL of volume can be assumed [17]. Once the meniscus obscures the hemidiaphragm, a volume of 500 mL of fluid is likely present [17]. The size of the effusion can also help guide the diagnosis of NMPE, and although in those with massive (those occupying most of the hemithorax) pleural effusion the etiology is typically malignancy, patients with cirrhosis and hepatic hydrothorax may present similarly [18]. Imaging using ultrasound is the standard for pleural fluid interventions, and pleural fluid acquisition is permissible when greater than 1 to 2 cm of pleural fluid separates the visceral and parietal pleura. 

**Figure 2:**
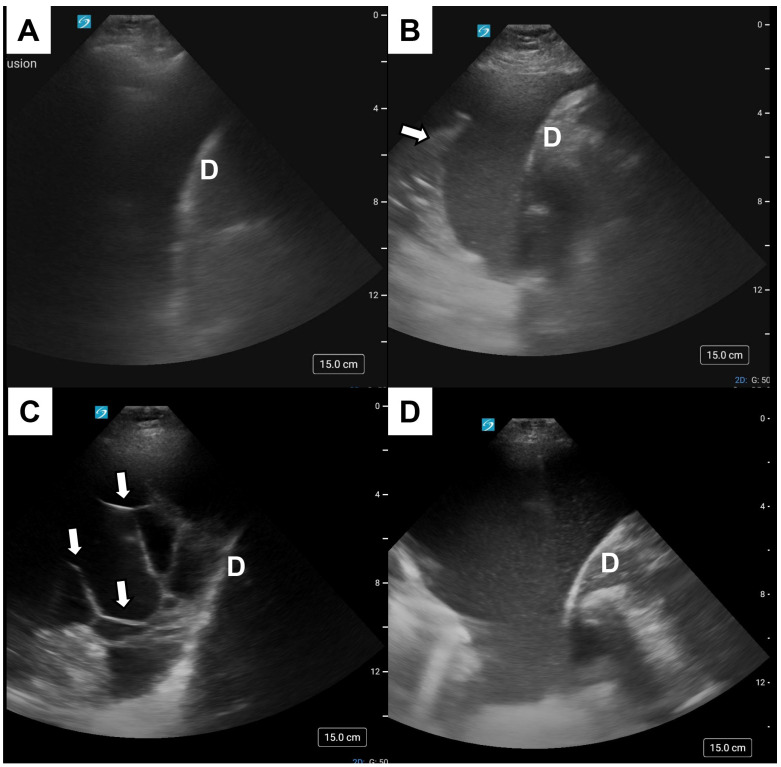
**Pleural ultrasound characteristics.** Ultrasound of the pleural space is categorized as simple (**A**) and complex (**B**–**D**). Categories of complex ultrasound include complex non-septated (**B**) noted with atelectatic lung (white arrow), complex-septated (**C**) with loculations (white arrows), and homogenously echogenic (**D**).

In current practice, pleural effusions are often incidentally detected on computed tomography (CT) of the chest or abdomen. Although CT does increase detection of pleural thickening, loculations, and/or increased density, the higher image quality is not useful in discriminating between transudates and exudates [19]. Ultrasound has emerged as an increasingly useful modality for pleural imaging due to its ease of use, portability, and cost-effectiveness [20]. Pleural ultrasound characteristics can be categorized as simple (or anechoic) or complex (hyperechoic) (Figure 2). Categories in the latter include complex non-septated (demonstrates swirling or floating echoes that appear light gray or white within the fluid correlating to protein, blood, or purulence); complex-septated (appears as strands within the effusion that can be free-floating or divide the effusion into discrete pockets); and homogenously echogenic (appears as uniform enhancement correlating to gelatinous purulence) [19]. Thoracic ultrasound is more sensitive than CT imaging in identifying complex effusions, which tend to be exudative [19]. In an evaluation of data from five series (560 transudates and 672 exudates), an anechoic sonographic pattern had a sensitivity of 80%, a specificity of 63%, a likelihood ratio (LR) positive of 2.16, and an LR negative of 0.32 for transudates [19]. Transudates in the complex category were mostly non-septated [21,22]. Due to these overlapping features, imaging cannot reliably discern between transudates and exudates; thus, biochemical sampling of pleural effusions of unknown etiology is paramount for further classification and workup [19,23].

Clinical correlation is also helpful in assessing the need for diagnostic pleural intervention. In cases with known organ dysfunction, conservative management is acceptable especially if pleural effusion resolves with treatment. The majority of transudative NMPE may be addressed with treatment of the underlying cause, but in those with refractory effusions, therapeutic interventions may be needed to alleviate symptoms as well as to exclude other etiologies. Serum biomarkers, such as N-terminal pro-brain natriuretic peptide (NT-proBNP), may be useful in those with heart failure and bilateral effusions, but in cases with an undiagnosed unilateral pleural effusion, sampling of pleural fluid may be needed as multiple conditions can co-exist [24]. Further, in those with suspicion of infection or malignancy, diagnostic pleural intervention should also be considered [24].

**Table 1:** Diagnostic testing for pleural effusions [12,13,14,15,16,25,26,27].

Test	Rule	Additional Comments
**Light’s Criteria**	PF_TP_/S_TP_ > 0.5 PF_LDH_/S_LDH_ > 0.6 PF_LDH_ > 2/3 normal S_LDH_	One of three rules in exudate SN: 98.3% SP: 76%
**Two-test Rule**	PF_LDH_ > 45% upper limit of normal S_LDH_ PF_cholesterol_ > 45 mg/dL	One of two rules in exudate SN: 98.1% SP: 69.4%
**Three-test Rule (Heffner’s Criteria)**	PF_TP_ > 2.9 g/dL PF_cholesterol_ > 45 mg/dL PF_LDH_ > 2/3 normal S_LDH_	One of three rules in exudate SN: 98.9% SP: 55%
**False Exudates**	S_albumin_–PF_albumin_ S_TP_–PF_TP_	Transudate if: Albumin gradient > 1.2 g/dL SN: 63% SP: 94% TP gradient > 3.1 g/dL SN: 86% SP: 81%
**Chylothorax**	PF_Triglycerides_ > 110 mg/dL PF_cholesterol_ < 200 mg/dL Chylomicrons present	Level between 50 mg/dL and 110 mg/dL does not exclude chylothorax; therefore, chylomicrons should be ordered
**Pseduochylothorax**	PF_Triglycerides_ < 50 mg/dL PF_cholesterol_ > 200 mg/dL Chylomicrons absent	
**Urinothorax**	PF_creatinine_/S_creatinine_ ratio > 1	Specificity improves with a higher ratio (range reported: 0.92–58)
**Bilothorax**	PF_bilirubin_/S_bilirubin_ ratio > 1	Evaluate for biliopleural fistula SN: 76.9

PF, pleural fluid; S, serum; TP, total protein; LDH, lactate dehydrogenase; SN, sensitivity; SP, specificity.

## 3. Cardiac-Related Etiologies

Cardiac etiologies for pleural effusions primarily include heart failure; however, effusions due to pericarditis, post-cardiac injury syndrome, and pulmonary hypertension may also occur [28]. Other causes may include effusions secondary to ischemic cardiomyopathy or non-ischemic etiologies such as valvular heart disease, restrictive heart disease, and hypertensive heart disease resulting in diastolic dysfunction.

***Heart failure.*** Heart failure-related pleural effusion has been associated with increased mortality, especially when compared with other etiologies of NMPE [29]. Specifically, in a prospective analysis of 356 NMPEs in a single center in England, effusions from heart failure had a 6-month and 1-year mortality of 40% and 50%, respectively [5]. Further, if the NMPE was bilateral (hazard ratio, HR, 3.55; 95% confidence interval, CI, 2.22–5.68) and transudative (HR 2.78, 95% CI 1.81–4.28), it carried a worse prognosis than being unilateral and exudative with a 1-year mortality of 57% and 43%, respectively [5]. This was corroborated in a prospective analysis in the United States as well [30]. Thus, NMPEs related to cardiac disease portend worse prognosis, likely reflecting suboptimal control of underlying disease.

The pathophysiology of pleural effusion in heart failure follows Starling’s principles. Increased hydrostatic pressure in the interstitial capillaries arises from elevation of left ventricular end-diastolic pressure and left atrial pressure, thus resulting in increased volume of interstitial fluid in the lung, which moves across the visceral pleura into the pleural space [28,31]. Fluid production overwhelms the lymphatic system’s ability to absorb and clear the pleural fluid, which may occur bilaterally or unilaterally. There are data to suggest that in patients with heart failure, lymphatic vessels may exhibit molecular and structural alterations, hindering drainage, and an inability to compensate for the excessive extravasation of fluid [32].

Symptoms of underlying heart failure may include breathlessness, fatigue, reduced exercise tolerance, ankle swelling, or less commonly nocturnal cough, wheezing, palpitation, dizziness, or bendopnea (shortness of breath when leaning forward) [33]. On exam, physical exam findings include elevated jugular venous pressure, hepatojugular reflux, third heart sound (gallop rhythm), laterally displaced apical impulse, peripheral edema (ankle, sacral, and scrotal), or ascites. In those with severe decompensation, patients may present with tachycardia, hypotension, or cold extremities [33].

The standard diagnostic imaging modality in patients with suspected heart failure is transthoracic echocardiography, which may show cardiomyopathy or valvulopathy. Specifically increased left atrial pressure can be reflected in an enlarged left atrium, increased mitral inflow velocity, and right ventricular systolic pressure. Those with an increased E/A ratio (early-to-late ventricular filling ratio) and increased right ventricular systolic pressure have a higher risk of developing pleural effusions [19]. Uncomplicated heart failure often has bilateral effusions in 73% of cases [6].

***Post-cardiac surgery.*** Pleural effusions after cardiac surgery can be seen in 10% of patients in the month following coronary artery bypass graft surgery, and in 25% of those patients, the pleural effusion will occupy more than 25% of the hemithorax [34]. Unilateral, small left-sided effusions can develop after cardiac surgery (up to 63% of patients post-coronary artery bypass), and these typically develop early and are related to trauma from surgery. Larger effusions may be divided into two categories based on time of occurrence (first 30 days and after 30 days) [35]. They are thought to be due to distinct processes. Early effusions are more likely to be bloody, eosinophil predominant and associated with high lactate dehydrogenase (LDH) and later effusions are characterized by a lymphocyte predominant, inflammatory-mediated process [35]. Pleural effusions may occur for a variety of reasons, including disruption of lymphatic channels, pleural injury, hypothermia, post-operative pericarditis, and post-cardiac injury (or Dressler’s syndrome). Most effusions spontaneously resolve or may require a single thoracentesis; however, non-steroidal anti-inflammatories can be given if Dressler’s syndrome is suspected [34].

***Pulmonary hypertension.*** Pulmonary hypertension in the absence of left heart disease has been associated with the development of pleural effusions, and it is an indicator of worse prognosis. Prevalence has been shown to vary significantly across different causes of pulmonary hypertension. The highest prevalence has been described in pulmonary hypertension secondary to connective tissue disease (39.3%), which may be related to the underlying inflammatory condition [36,37]. In one single-center study of patients with pulmonary arterial hypertension (excluding those with left heart disease, lung disease, and chronic thromboembolic disease), pleural effusion not attributable to inflammation or other causes was found in 7.3% of patients, with the majority (59.0%) having bilateral pleural effusions [38]. These patients were more likely to have elevated right atrial pressure and lower body mass index, as well as attenuated survival independent of pericardial effusion. The mechanism resulting in pleural effusion remains unclear, and isolated right atrial and ventricular pressures and/or other possible mechanisms may increase capillary permeability or impair lymphatic clearance [36,37,39].

***Management***. Complete resolution of pleural effusions with diuretics may require several weeks, but a large proportion will improve when treatment for heart failure is optimized. In a prospective study of 60 patients, 89% no longer had a pleural effusion after initial diuretic treatment at follow-up after 2 weeks [40]. However, up to 25% of pleural effusions may not resolve with diuresis alone [41].

Current guidelines and randomized trials supporting the use of thoracentesis for cardiac related pleural effusions are limited, and more data are needed to assess the outcomes associated with thoracentesis, chest tube insertion, and tunneled indwelling pleural catheter (TIPC) placement. Shetty and associates analyzed data from a nationwide inpatient sample and found higher rates of in-hospital mortality in heart failure patients undergoing thoracentesis [42]. Glargaard and colleagues recently concluded an open-label, randomized controlled trial (TAP-IT) that evaluated outcomes such as mortality, complications, readmissions, and quality of life in patients undergoing thoracentesis while receiving medical therapy versus medical therapy alone, but data are pending release [43]. 

Despite the approval of the use of TIPCs for the management of refractory NMPEs by the Food and Drug Administration, there is a lack of data regarding their safety in patients with heart failure-related pleural effusions. In a case series published by Herlihy and colleagues, the use of TIPCs in heart failure-related pleural effusions was associated with a high complication rate [44]. The REDUCE trial prospectively compared repeated thoracentesis versus TIPCs in NMPEs (n = 68, with 46 related to cardiac dysfunction), and the primary outcome was mean daily dyspnea score. There was no statistically significant difference in symptoms between the two groups, and TIPCs were not superior to repeated thoracentesis in their cohort. Furthermore, the TIPC arm had more adverse outcomes, including pleural space infection and cellulitis [45]. On the contrary, a meta-analysis published by Patil and co-authors had different findings in an analysis of 13 studies and a total of 325 patients with NMPE who received a TIPC. Approximately 50% had NMPE related to heart failure, and they reported a lower rate of minor complications, reduction in hospital stay, and readmission rates when compared to patients who did not receive a TIPC. Additionally, 42.1% of patients achieved pleurodesis, demonstrating that perhaps there is a role for TIPC placement in refractory benign pleural effusions; however, the quality of evidence remains low [46]. We would advocate for management decisions on a case-by-case basis in a multi-disciplinary team and shared decision making with the patient and their family.

There are currently no robust data that evaluate the efficacy and outcomes of chemical or surgical pleurodesis in patients with NMPE related to cardiac dysfunction, but there are two retrospective studies. Freeman and associates compared talc pleurodesis with TIPC in patients with recurrent pleural effusions secondary to heart failure, and although both methods provided similar palliation (lack of re-intervention during follow-up and improvement in performance scores), TIPC resulted in a significantly shorter stay and lower rates of operative readmissions and morbidity in comparison to talc pleurodesis [47]. Majid and associates evaluated the utility of TIPCs with and without talc poudrage in heart failure-related pleural effusions. The success rate of pleurodesis in the talc poudrage group was 80%, compared to 25% in catheter placement alone, and it reduced the days to catheter removal. However, this was a retrospective study, and TIPC-related complications occurred in both groups (infection and cellulitis) [48]. Pleuro-peritoneal or pleuro-venous shunting of pleural fluid from the pleural space for NMPE has been described in case series, but this technique is contraindicated in patients with ascites [19]. Surgical approaches are typically avoided due to frailty and other comorbidities [19].

## 4. Liver Dysfunction

Hepatic hydrothorax is a consequence of end-stage liver disease and is associated with poor prognosis. It occurs in 5–15% patients with cirrhosis and portal hypertension. It is characterized by a transudative effusion that develops in the absence of cardiac disease [49]. The presence of hepatic hydrothorax is a significant indicator of mortality [50]. Ascites accumulates in the peritoneal cavity and migrates down the pressure gradient into the negative intrathoracic space through small diaphragmatic defects [51]. These defects are more commonly found in the right hemidiaphragm, leading to a disproportionate number (80%) of right-sided hepatic hydrothorax, but left-sided pleural effusion may also occur [5]. The recently published CIRrhotic Ascites Severity (CIRAS) model, which relies only on ascites-associated information, stratifies patients at different risks for the development of hepatic hydrothorax and outperforms Child–Pugh and MELD in predicting the first occurrence of hepatic hydrothorax requiring thoracentesis within one year of follow-up [52].

Management of hepatic hydrothorax is challenging, with approximately 25% of cases refractory to medical therapies [53]. Repeated thoracentesis has a higher rate of complications, and chest tube insertion followed by talc pleurodesis is often unsuccessful due to rapid fluid accumulation. Options for treatment include medical management of ascites, transjugular intrahepatic portosystemic shunts (TIPSs) for refractory disease, liver transplant, or pleural interventions. Liver transplant is the only curative treatment [19]. TIPS is estimated to lead to a partial resolution in 55.8% of cases [54,55]. In both case series and retrospective studies, TIPC carries a significant risk for infection, and it may have adverse sequelae due to intravascular volume loss [56,57]. TIPC insertion achieves pleurodesis in approximately 17% cases with an average pleural infection rate of 12.5% over 4.4 months [57]. In these cases, a multi-disciplinary approach within the patient’s respective center is recommended.

## 5. Renal-Related Etiology

Renal disease can manifest with pleural complications from a variety of causes. Most commonly, end-stage renal disease (ESRD) patients will present with pleural effusions due to hypervolemia or underlying cardiac disease [58]. However, care must be taken to consider less common alternatives as well as infection and malignancy [19].

Patients with nephrotic syndrome may have pleural effusions due to low oncotic pressure (proteinuria) and increased hydrostatic pressure (salt retention). Effusions are typically transudative but may be exudates as well. These patients are also more hypercoagulable due to protein loss, as well as more susceptible to infection from loss of immunoglobulins [59]. Treatment is directed towards management of nephrotic syndrome as well as interventions to optimize fluid overload and hypoproteinemia [19].

Uremic pleuritis is typically a diagnosis of exclusion, and chronic fibrinous pleuritis is noted on pleural biopsy [60,61]. Although the mechanism is unknown, it is suggested that toxins from uremia lead to effusion or bleeding from coagulopathy. Moreover, the use of heparin may also cause effusion. Management may include increasing the intensity of renal replacement therapy, chest tube placement with chemical pleurodesis, pleural decortication or potentially systemic steroids [19].

Peritoneal dialysis (PD) can be complicated by pleuro-peritoneal leak, and translocation is caused by increased intra-abdominal pressure leading to transdiaphragmatic migration and pleural effusion formation. Pleuro-peritoneal leak is easily recognized by pleural fluid analysis, which demonstrates a transudate with extremely low protein levels (<1 g/dL) and elevated glucose levels (350–450 mg/dL) due to dialysate [62]. Management includes using an alternative mode of renal replacement therapy, pleurodesis, and surgical repair [19].

Vascular abnormalities secondary to complications from hemodialysis may also occur. For example, an arteriovenous fistula creation may lead to increased hydrostatic pressure due to vascular obstruction, resulting in unilateral transudative pleural effusion [63]. Venography can be diagnostic, and ligation of the fistula or venoplasty is curative [19].

Urinothorax results from obstructive uropathy or trauma of the urinary system leading to transdiaphragmatic travel of urine into the pleural cavity [59]. Diagnosis necessitates a high clinical suspicion, and pleural effusion can be transudative or exudative. Nuclear medicine study with renal scintigraphy can detect extravasation of the trace dye. Once diagnosed, surgical or radiological intervention for anatomical defect is often needed [19].

Management strategies in addition to adjustments in renal replacement therapy have included limited trials evaluating TIPC placement in ESRD patients with recurrent effusions. Potechin and associates reported a 37.5% rate of auto-pleurodesis and significant improvement in symptoms with a lack of major complications [64]. Pleurodesis has been evaluated in the PD population who experience pleuro-peritoneal leak. Tube thoracostomy-directed pleurodesis in one series was successful in 48% of cases [65]. Video-assisted thoracic surgery assisted pleurodesis with either mechanical or talc pleurodesis demonstrated a 90% success rate in a series by Chen and colleagues [7]. Diaphragmatic repairs have also been performed in this group with reported success rates up to 100% [66]. 

## 6. Other Organ Dysfunctions

Beyond the common etiologies, NMPE can also arise from a diverse range of less frequent causes.

***Sarcoidosis.*** Pleural effusion is a rare finding in pulmonary sarcoidosis and is typically found early in the disease course in the absence of medical therapy due to active granulomatous inflammation [63]. Effusions are often small, unilateral, and exudative with a lymphocytic predominance. Corticosteroids are highly effective, and effusions should resolve by 4–6 weeks [67].

***Systemic Lupus Erythematosus***. Lupus pleuritis can occur in up to 60% of patients, typically coexisting with other organ diseases [68]. Typical pleural fluid findings include a clear exudative effusion with positive pleural fluid ANA and leukocytes with normal glucose. Non-severe cases can be managed with non-steroidal anti-inflammatories and corticosteroids for severe or refractory cases [69]. Lupus pleuritis may progress to fibrothorax and trapped lung [70].

***Rheumatoid Arthritis***. Pleural effusions occur in up to 20% of patients with rheumatoid arthritis, mostly in men [71]. The pleural fluid profile is notable for exudative effusion with low glucose (<30 mg/dL), leukocytosis, and positive rheumatoid factor [71]. The fluid appearance can be variable—from serous, bloody, and purulent to milky. Most effusions will resolve with systemic therapy; however, large refractory effusions may progress to fibrothorax and trapped lungs without drainage [70].

***IgG4-related disease***. An uncommon cause of lymphocytic, exudative effusion is IgG-4-related disease, which is an immune-mediated condition. This can masquerade as a tuberculous effusion due to its predilection to also have positive pleural fluid adenosine deaminase (ADA) [72]. This can be an important cause of otherwise unexplained exudative effusions. Concomitant pleuritis or pleural masses may be present. Diagnosis requires pleural biopsy with positive staining for IgG4 with plasma cell infiltration. Treatment consists of corticosteroids [73].

***Pulmonary embolism.*** Often a small unilateral pleural effusion accompanies acute PE. Pleural fluid is exudative and often bloody. Pleural fluid eosinophilia is also seen [74]. Without intervention, pleural fluid will be reabsorbed after initiation of anticoagulation after approximately one week but can take up to 3 weeks in those with evidence of pulmonary infarction [75].

***Post-Surgical–Related.*** Abdominal surgery can also lead to unexpected pleural effusions. Up to 50% of patients may develop pleural effusion shortly following abdominal surgery, which typically resolves spontaneously [76].

***Chylothorax and pseudochylous effusions.*** A chylothorax is characterized as a milky appearing, often unilateral pleural effusion. It occurs because of a chyle leak into the pleural space due to an injury to the thoracic duct. Approximately half of cases are related to trauma (penetrating chest and surgical instrumentation), and half are non-traumatic (lymphoma, superior vena cava syndrome, lymphangiomatosis, and granulomatous infections) [77].

Diagnosis is made by pleural sampling (Table 1). For pleural fluid triglyceride levels between 50 and 110 mg/dL, chylomicron analysis can be performed, or repeat pleural sampling after a high-fat meal can also be considered [78].

Management for those related to trauma consists of a low-fat diet or trial of total parenteral nutrition. It may take up to two weeks to see improvement [8]. For refractory effusions, lymphangiography may be performed to identify the site of injury with eventual thoracic duct embolization, which has a success rate of up to 90% in traumatic cases and 50% in non-traumatic [79]. In those related to lymphoma, treatment of malignancy and TIPC are used, and no increased risk of infection or protein loss has been reported [80].

Pseudochylous effusions are also often unilateral with a milky appearance like chylothorax. However, pleural fluid analysis will demonstrate a preponderance of cholesterol with no chylomicrons. Pseudochylous effusions result from a chronic exudative effusion that slowly develops elevated levels of cholesterol due to lack of transfer from the pleural space. Causes include infections (such as tuberculosis, echinococcus, and paragonimiasis), rheumatoid arthritis, or malignancy [81].

### 6.1. Gastrointestinal Disease Related

Gastrointestinal diseases can lead to pleural effusions through direct extension, transdiaphragmatic spread, or systemic inflammatory responses.

***Esophageal perforation.*** Pleural fluid is exudative, highly acidic (pH < 7), and often with elevated amylase levels. If untreated, it can lead to empyema. Often in this setting there is coexistent mediastinitis and sepsis due to rupture. A definitive diagnosis can be made by CT imaging [82].

***Pancreatic Disease.*** As many as 50% of patients can present with pleural effusions related to acute pancreatitis, which result from the diaphragmatic inflammation and transdiaphragmatic transfer of the exudative fluid arising from acute pancreatic inflammation. Although typically small, exudative, and left-sided, they are a marker of disease severity and carry a mortality rate up to 30% [9].

Chronic pancreatitis can result in a pancreatico-pleural fistula due to injury of the main pancreatic duct. The effusion has a very high pleural fluid amylase level—generally greater than 1000 U/L [83]. Diagnosis is made by using magnetic resonance cholangiopancreatography (MRCP). Closure can be performed conservatively by medical treatment of chronic pancreatitis or endoscopic/surgical procedures [84].

***Bilious pleural effusion***. Bile can enter the pleural space through several mechanisms, including percutaneous transhepatic biliary drainage, complications of biliary infections, or trauma. Generally, the effusion is right-sided, and pleural fluid analysis will demonstrate a pleural fluid to serum bilirubin ratio of greater than 1.0. Fluid appears bile-colored or black [85]. Resolution requires management of the source of biliary obstruction or leak.

### 6.2. Gynecology-Related Conditions

Gynecologic conditions can cause pleural effusions through mechanisms like transdiaphragmatic spread, malignancy, or hormonal influences.

***Meigs syndrome***. Meigs syndrome is defined by the constellation of pleural effusions, ascites, and an ovarian fibroma. The effusion is commonly right-sided and unilateral. Pleural fluid analysis generally indicates an exudative effusion. The mechanism for pleural accumulation is thought to result from transfer of ascites from the peritoneal cavity via the diaphragm [86]. Following tumor resection, ascites and pleural effusion typically resolve over several weeks.

***Endometriosis*.** Bloody or chocolate-colored right-sided exudative effusions can be caused by severe endometriosis. Often these effusions will be moderate to large and present with coexistent ascites. Pneumothorax may develop before, after, or synchronously with the pleural effusion [87]. Pleural fluid carcinoembryonic antigen level and other tumor markers should be tested to evaluate for ovarian cancer. Management can be complex, requiring a combination of hormonal therapy and thoracic and abdominal surgeries in up to 52% of patients [88].

***Ovarian hyperstimulation syndrome (OHSS).*** OHSS is a complication of assisted reproductive technologies, characterized by increased vascular permeability leading to fluid shifts and third-spacing, including pleural effusions. The pathophysiology involves elevated vascular endothelial growth factor (VEGF), which promotes capillary leakage and fluid accumulation in pleural and peritoneal spaces [89]. Pleural effusions in OHSS are typically right-sided and associated with ascites, hemoconcentration, and thromboembolic risks [90]. Management is primarily supportive, with fluid balance optimization and, in severe cases, thoracentesis for symptomatic relief [91].

## 7. Drug-Related Pleural Disease [92]

Drug-induced pleural effusions (Table 2) are important to recognize, as they can mimic other causes of pleural disease. These effusions are typically exudative and may be associated with eosinophilia, lymphocytosis, or, in some cases, a drug-induced lupus-like syndrome [93]. The mechanism varies by drug and can include direct pleural irritation, hypersensitivity reactions, immune-mediated inflammation, or drug-induced lupus [10]. Management involves identifying and discontinuing the offending drug, which usually leads to resolution. In severe cases, corticosteroids or drainage may be needed.

## 8. Future Directions and Conclusions

NMPEs are frequently encountered in various disease states (Table 3). Future research for both diagnostic and management strategies for NMPE is needed. Additional information on patient-centered outcomes, such as quality of life and symptom assessment, would also be helpful in terms of management. Systemic workups to identify NMPE and optimize the underlying etiology can ameliorate both symptoms and the effusion.

## Figures and Tables

**Table 2:** Drug-induced pleural disease [10,93].

	**Examples**	**Proposed Mechanism of Pleural Effusion**
**Medications causing drug-induced lupus (DIL)**	Hydralazine, procainamide, and isoniazid	▪ Immune complex deposition in pleura ▪ Usually presents with other lupus-like symptoms
**Antibiotics**	Nitrofurantoin	▪ Hypersensitivity reaction or eosinophilic infiltration of pleura
**Antiarrhythmic**	Amiodarone	▪ Accumulation of drug/metabolites in the pleural space ▪ May cause lipid-laden macrophages in pleural fluid
**Non-steroidal anti-inflammatory drugs (NSAIDs)**	Ibuprofen, Diclofenac	▪ Hypersensitivity reaction, increased capillary permeability ▪ Can also be associated with drug-induced lupus
**Biologics (TNF-alpha inhibitors)**	Infliximab	▪ Immune-mediated reaction ▪ May be associated with autoimmune pleuritis
**Chemotherapeutic agents**	Methotrexate, bleomycin, dasatinib, and gemcitabine	▪ Direct toxicity ▪ Often associated with drug-induced pneumonitis ▪ Immune-mediated

**Table 3:** Summary of non-malignant pleural effusion etiologies, pathophysiologic mechanisms, and management strategies [7,8,9,18,36,42,51,59,63,68,73,75,76,77,78,80,81,82,85].

**Etiology**	**Mechanism**	**Management**
**Cardiac**		
Cardiac-related	Increased hydrostatic pressure and impaired lymphatic drainage	▪ Diuresis ▪ Thoracentesis for symptomatic relief ▪ Limited role for chest tube insertion or TIPC
Post-cardiac surgery	Disruption of lymphatic channels, pleural injury, hypothermia, post-operative pericarditis, and post-cardiac injury (or Dressler’s syndrome).	▪ Thoracentesis if unresolved ▪ Non-steroidal anti-inflammatories if Dressler’s syndrome is present
Pulmonary hypertension	Elevated right heart pressure may increase capillary permeability or impair lymphatic clearance	▪ Diuresis ▪ Medical management of pulmonary hypertension
**Hepatobiliary**		
Hepatic hydrothorax	Ascitic fluid migration via diaphragmatic defects	▪ Medical management of ascites ▪ Transjugular intrahepatic portosystemic shunt (TIPS) for refractory cases ▪ TIPC in select cases ▪ Liver transplant
Bilious effusion	Bile leakage from the biliary system into pleura occurs because of percutaneous trans-hepatic biliary drainage, complications of biliary infections, or trauma	▪ Pleural drainage and treatment for underlying biliary disease
**Renal and Genito-urinary**		
Renal-related	Hypervolemia, nephrotic syndrome, uremia, and peritoneal dialysis leakage	▪ Treating underlying renal dysfunction and thoracentesis for symptomatic relief, if needed ▪ Pleuro-peritoneal leak managed by using alternative modes of renal replacement therapy or surgical repair ▪ TIPC in select refractory cases
Urinothorax	Urologic process resulting in a trans-diaphragmatic translocation of urine into the pleural cavity	▪ Pleural drainage and primary management of urologic diseases
**Chylous and Pseudochylous**		
Chylothorax	Chyle leakage due to thoracic duct injury (trauma, lymphoma, superior vena cava syndrome, and infections)	▪ Low-fat diet or TPN ▪ Thoracic duct embolization (90% success in trauma and 50% in non-trauma) ▪ TIPC for malignant etiologies
Pseudochylous	Chronic exudative effusion with cholesterol accumulation	▪ Treating underlying cause (e.g., TB, RA, and malignancy)
**Gastrointestinal**		
Esophageal perforation	Direct leak of esophageal contents into pleura	▪ Urgent surgical repair ▪ Pleural drainage and antimicrobials
Pancreatic disease	Transdiaphragmatic spread of inflammatory fluids	▪ Supportive care for acute pancreatitis ▪ Octreotide and/or endoscopic closure for pancreatico-pleural fistulas
**Gynecological**		
Meigs Syndrome	Transfer of ascitic fluid across the diaphragm in ovarian fibroma	▪ Surgical resection of a tumor
Endometriosis	Ectopic endometrial tissue in the thoracic cavity	▪ Hormonal therapy and/or surgery (thoracic and abdominal procedures)
Ovarian Hyperstimulation Syndrome	Increased vascular permeability from high VEGF levels	▪ Supportive care, fluid balance management, and thoracentesis for severe cases
**Autoimmune and Inflammatory**		
Sarcoidosis	Found in early disease due to active granulomatous inflammation	▪ Corticosteroids
Systemic lupus erythematosus (SLE)	Immune complex-mediated pleural inflammation (lupus pleuritis)	▪ NSAIDs for mild cases ▪ Corticosteroids for severe/refractory cases
Rheumatoid arthritis	Chronic inflammation	▪ Systemic therapy ▪ Drainage for large refractory effusions to prevent fibrothorax
IgG4-related	Immune-mediated inflammation	▪ Corticosteroids

TIPC, tunneled indwelling pleural catheters; TPN, total parenteral nutrition; TB, tuberculosis; RA, rheumatoid arthritis; VEGF, vascular endothelial growth factor; NSAIDs, non-steroidal anti-inflammatories.

## Data Availability

All data collected in this study are provided in this paper.

## Abbreviations

The following abbreviations are used in this manuscript:

NMPE	Non-malignant pleural effusion
CT	Computed tomography
LR	Likelihood ratio
NT-proBNP	N-terminal pro-brain natriuretic peptide
PF	Pleural fluid
S	Serum
LDH	Lactate dehydrogenase
HR	Hazard ratio
CI	Confidence interval
E/A ratio	Early-to-late ventricular filling ratio
TIPC	Tunneled indwelling pleural catheter
TIPS	Transjugular intrahepatic portosystemic shunt
ESRD	End-stage renal disease
PD	Peritoneal dialysis
ADA	Adenosine deaminase
MRCP	Magnetic resonance cholangiopancreatography
OHSS	Ovarian hyperstimulation syndrome
VEGF	Vascular endothelial growth factor
